# Peripartum HFpEF: Identification of a Novel Phenotype and the Need for Cardio-Obstetrics Management

**DOI:** 10.1016/j.jacadv.2023.100799

**Published:** 2024-01-12

**Authors:** Salva Yurista, Priya Wadhera, Robert A. Eder, Uri Elkayam, Omar K. Siddiqi

**Affiliations:** a Section of Cardiovascular Medicine, Department of Medicine, Boston Medical Center, Boston University Chobanian and Avedisian School of Medicine, Boston, Massachusetts, USA; b New York Institute of Technology College of Osteopathic Medicine, New York, New York, USA; c Division of Cardiovascular Medicine and Department of Obstetrics and Gynecology, Department of Medicine, University of Southern California, Los Angeles, California, USA

**Keywords:** heart failure, HFpEF, peripartum cardiomyopathy

The landscape of women's health has been profoundly impacted by the rising prevalence of cardiovascular disease, which now stands as the leading cause of death among pregnant women and those in the postpartum period. Recent data highlight a concerning trend, with cardiovascular conditions accounting for a significant 26.5% of pregnancy-related deaths in the United States.^1^^,^^2^ These numbers serve as a stark reminder of the critical importance of enhancing our understanding, prevention, and management of cardiovascular health during and after pregnancy. It is imperative that we prioritize the well-being of women by implementing comprehensive strategies to address this pressing issue and ensure optimal care for expectant and new mothers. In this viewpoint, we will explore the question of whether elevated natriuretic peptides in pregnant and postpartum patients with preserved left ventricular (LV) ejection fraction and clinical signs of heart failure (HF), found in late pregnancy or early after delivery, represent a novel, poorly understood phenotype of HF associated with pregnancy, distinct from peripartum cardiomyopathy (PPCM). Furthermore, we discuss whether follow-up cardiology evaluations are necessary for such patients. While these patients may initially appear to have normal left ventricular systolic function, the presence of elevated natriuretic peptides raises concerns about potential underlying cardiac abnormalities. By examining the available evidence, clinical considerations, and potential benefits and risks, we will discuss the rationale and potential implications of postdischarge cardiology follow-up for this specific patient population.

## PPCM: a brief primer

The field of cardio-obstetrics is rapidly emerging as a vital discipline in healthcare. To date, the American Heart Association (AHA), American College of Cardiology (ACC), Heart Failure Society of America (HFSA), and the European Society of Cardiology (ESC), as well as the American College of Obstetricians and Gynecologists (ACOG), acknowledge that PPCM is a rare and potentially life-threatening condition characterized by HF resulting from left ventricular systolic dysfunction with a left ventricular ejection fraction (LVEF) below 45%. PPCM typically occurs during the final month of pregnancy or in the months following delivery, with a higher incidence observed within the first month postdelivery. It is important to note that PPCM is a diagnosis of exclusion, requiring the absence of any other identifiable cause of HF in these cases.^3^, ^4^, ^5^, ^6^ The exact cause of PPCM remains unclear, but it is believed to involve a combination of various factors. The “two-hit” hypothesis proposes that changes in cardiovascular adaptation during pregnancy (the first hit) along with additional stressors (the second hit) can contribute to the development of PPCM. Further research is needed to fully understand the underlying mechanisms of PPCM and identify the specific factors involved.^3^ Additionally, epidemiological studies have shown that certain factors increase the likelihood of developing PPCM, including advanced maternal age, multiparity (having multiple pregnancies), African descent, pre-existing hypertension or preeclampsia, multiple gestations (such as twins or triplets), obesity, and a history of PPCM in previous pregnancies. Of note, preeclampsia is strongly linked to an increased predisposition to PPCM, likely due to shared underlying pathophysiological mechanisms.^2^^,^^3^^,^^5^

**Figure 1:**
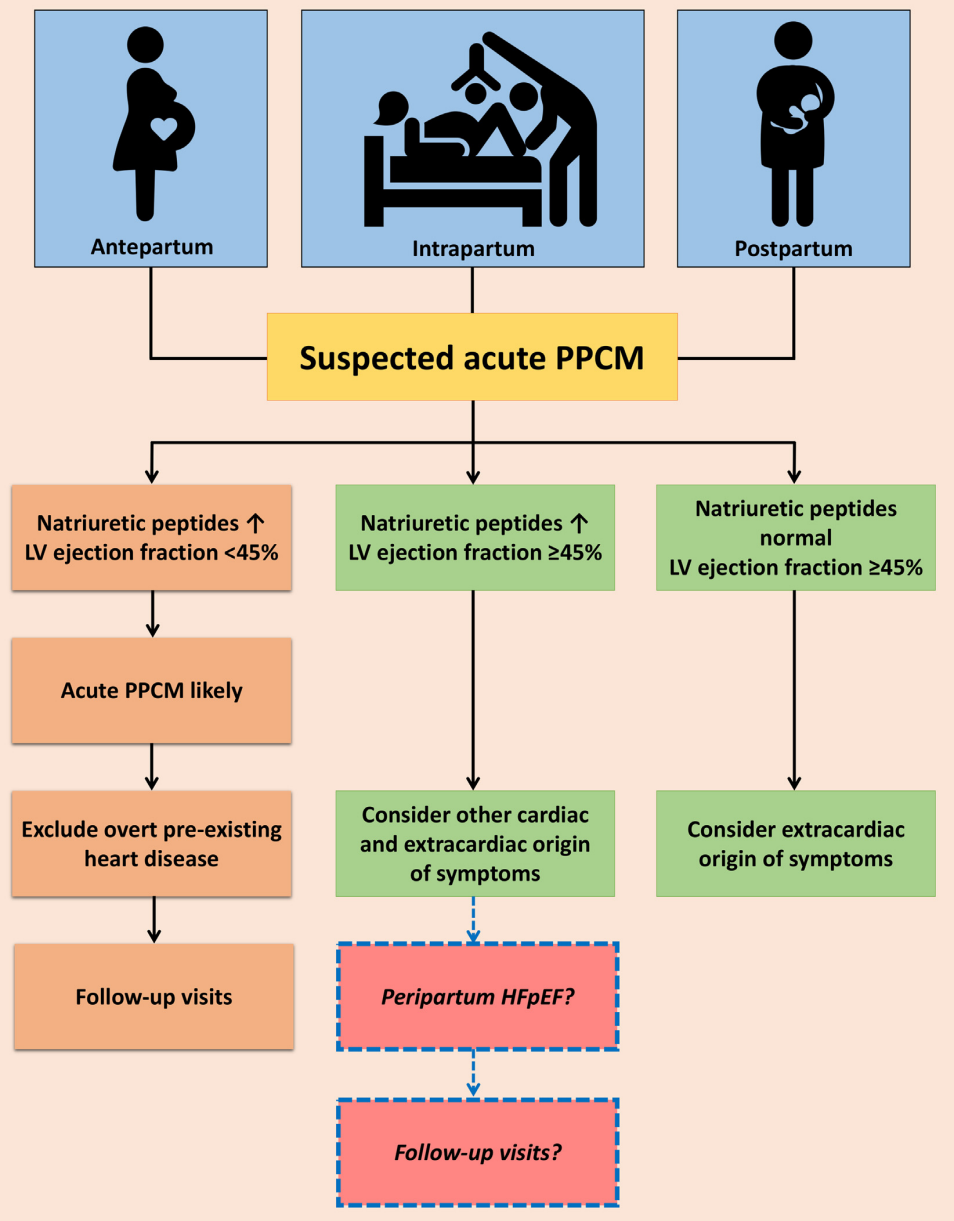
**Diagnostic Pathway for Peripartum Cardiomyopathy and the Need for Cardio-Obstetrics Follow-Up** The figure illustrates the diagnostic pathway for peripartum cardiomyopathy (PPCM) based on the ESC working group's recommendations. It prompts the exploration of potentially new phenotypes of peripartum heart failure and the importance of follow-up visits for patients who display elevated natriuretic peptides alongside preserved ejection fraction. This underscores the need for further research and the development of clearer recommendations to address these aspects. Adapted from Bauersachs et al. *Eur J Heart Fail*. 2019;21(7):827-843.

Diagnosing and managing PPCM remains challenging, as it is often a diagnosis of exclusion despite the existence of several guidelines.^2^, ^3^, ^4^, ^5^ The diverse range of presentations, varying from subtle signs to severe manifestations like acute HF, pulmonary edema, or cardiogenic shock, further complicates the management of PPCM.^3^ This emphasizes the critical need for increased awareness and physician education to allow for early recognition and accurate diagnosis to facilitate timely and appropriate interventions. ACOG recommends all pregnant or postpartum women who experience symptoms such as shortness of breath, chest discomfort, palpitations, arrhythmias, or fluid retention undergo evaluation for PPCM.^2^ Diagnostic testing for PPCM includes a combination of clinical assessment, laboratory tests, and imaging studies (Figure 1). Clinical evaluation involves a thorough medical history, physical examination, and assessment of symptoms. ESC recommends performing an electrocardiogram in all patients with suspected PPCM as it is a safe and cost-effective diagnostic tool that can aid in distinguishing PPCM from other causes of symptoms; however, it is important to note that there is no specific electrocardiogram pattern that is diagnostic of PPCM.^3^

Blood tests in symptomatic patients should include measuring natriuretic peptides, such as brain natriuretic peptide (BNP) or N-terminal pro-B-type natriuretic peptide (NT-proBNP), which are often elevated in PPCM. It should be noted that normal pregnancy is not usually associated with significant or pronounced elevation of B-type natriuretic peptide BNP and NT-proBNP levels. According to the ESC recommendation, the main utility of natriuretic peptides is to help rule out HF. A BNP level below 100 pg/mL and an NT-proBNP level below 300 pg/mL provide a high probability of ruling out HF. These cutoff values serve as a useful reference in clinical practice to help differentiate patients who are less likely to have HF. However, it’s worth emphasizing that these cutoff values may not be specific to PPCM and should be interpreted in the context of the overall clinical presentation and diagnostic evaluation.^3^ In a subset of patients with HF, especially those who are obese (a group strongly associated with PPCM),^7^ some may exhibit normal BNP levels. In such cases, it is advisable to consider ordering an echocardiogram primarily for patients displaying signs and symptoms of HF, even when BNP levels remain normal. Additional blood tests may be performed to help exclude other potential causes and aid in the differential diagnosis of PPCM. Imaging studies, particularly echocardiography, play a key role in diagnosing PPCM by assessing LV systolic function, evaluating chamber dimensions, and identifying any structural abnormalities. Other imaging modalities, such as cardiac magnetic resonance imaging, may be employed in certain cases to provide further characterization of cardiac function and tissue characteristics. The combination of these diagnostic tests aids in confirming the diagnosis of PPCM and guiding appropriate management strategies.^2^^,^^3^^,^^6^

According to the guidelines of AHA/ACC/HFSA for the management of HF, the treatment of HF during pregnancy is centered around managing volume status through the administration of diuretics, reducing afterload by prescribing medications like nitrates and hydralazine, controlling rhythm using beta-blockers and digoxin, and considering the use of anticoagulation when necessary.^6^ The use of pharmacological blockade of prolactin release using bromocriptine in conjunction with standard HF treatment, as it has shown promising results in 2 clinical trials as a potential therapy for PPCM.^3^ These therapeutic interventions aim to optimize cardiac function and support maternal well-being during this critical period.

The prognosis for PPCM can vary from person to person. While some women may experience complete recovery of their heart function, others may continue to have symptoms or may even see their condition worsen over time. Among the various prognostic factors studied, baseline ejection fraction at the time of diagnosis is recognized as the most reliable predictor of adverse events or long-term recovery in PPCM. Additional predictors of a worse outcome include the presence of LV dilatation, LV thrombus, right ventricular systolic dysfunction, and obesity. However, with appropriate medical treatment, close monitoring, and adherence to lifestyle modifications, many women with PPCM can achieve significant improvement in their cardiac function and have a positive long-term outlook. Regular follow-up visits and ongoing medical management are essential to optimize prognosis and prevent complications in women with PPCM.^3^^,^^5^

## Outstanding questions: a viewpoint

The ESC study group has provided a helpful diagnostic pathway for PPCM. This pathway takes into consideration the levels of natriuretic peptides and the LVEF to guide the evaluation of patients with suspected PPCM.^3^ Firstly, elevated natriuretic peptides and a decrement in LVEF to <45% suggest acute PPCM. Secondly, if the natriuretic peptides are elevated but the LVEF is 45% or higher, other potential causes of HF with preserved ejection fraction (HFpEF) or HF with mildly reduced ejection fraction should be considered. Thirdly, if the natriuretic peptides are normal and the LVEF is 45% or higher, noncardiac causes of symptoms should be explored.^3^

In the field of cardiology, it is not uncommon to receive consultation requests from obstetricians to evaluate patients with suspected PPCM. However, a challenging situation arises when patients meet the second criteria outlined by ESC. These patients exhibit signs and symptoms along with elevated levels of natriuretic peptides while maintaining normal LVEF as measured by echocardiography or cardiac magnetic resonance imaging. In this category, ESC advises conducting additional examinations to differentiate potential differential diagnoses. These may include investigating conditions like pulmonary embolism, amniotic fluid embolism, isolated right ventricular dysfunction, hypertensive disorders of pregnancy, eclampsia, and sepsis.^3^ However, it becomes more challenging when there is no clear alternative explanation for the elevated BNP levels, classic symptoms of acute HF without LV systolic dysfunction in the absence of pre-eclampsia, eclampsia, or hypertension.

*While the ESC study group has compiled a detailed statement on PPCM, significant uncertainties persist regarding the optimal management of patients who present with HF symptoms, elevated natriuretic peptides, and normal LVEF (≥45%), as opposed to traditionally defined PPCM (LVEF <45%).* This unique and potentially novel phenotype within the spectrum of PPCM requires dedicated attention and investigation. By recognizing and distinguishing this distinct subset of patients with elevated natriuretic peptides but normal echocardiogram findings, we can gain invaluable insights into the underlying mechanisms, clinical implications, and optimal management strategies specific to this phenotype. It is essential to emphasize the significance of identifying and studying this phenomenon, as it has the potential to impact prognosis, therapeutic approaches, and long-term outcomes for affected individuals.

One other key question is whether routine follow-up is necessary for this specific patient population who exhibit classic symptoms of HF with increased natriuretic peptide levels and LVEF below 45% who fall into the second category. Determining the optimal duration and frequency of follow-up visits is also crucial for effective monitoring and timely intervention. Additionally, it is important to identify specific risk factors or clinical features that can help identify patients at higher risk of disease progression or adverse outcomes. Furthermore, understanding the long-term outcomes and prognosis for these patients is essential for providing appropriate care and support. Unfortunately, the current guidelines and statements from cardiology societies such as ESC and ACC/AHA/HFSA do not provide sufficient answers to these outstanding questions.

Given the timing of PPCM development during the last month of pregnancy or within 5 months postdelivery, based on our experiences, it may be important to consider clinical cardiology follow-up once patients are discharged. Although patients may have normal echocardiogram findings initially, there is evidence to suggest that changes in LVEF and left ventricular dimensions occur acutely around the time of or shortly before the diagnosis of PPCM. A small study reported that LVEF and LV dimensions were largely normal during pregnancy and the early postpartum period in women who later developed PPCM. However, subclinical strain abnormalities, as measured by global longitudinal strain (GLS) and global circumferential strain (GCS), were identified during pregnancy in these women.^8^ This suggests that incorporating subclinical strain measurements in women at higher risk of developing PPCM during routine pregnancy assessments may provide valuable information for early diagnosis and risk assessment. That stated, further research is needed to fully understand the clinical significance of subclinical strain abnormalities and to establish the optimal follow-up strategies in this population. In addition, the occurrence of acute postpartum HF with preserved systolic function has been reported previously, highlighting case where this condition was observed in the absence of pre-eclampsia or prior cardiovascular disease. Despite uncertainties, it remains unclear whether the described case represents the mildest form of the PPCM spectrum or constitutes a distinct form of diastolic HF.^9^

Next, optimal follow-up strategies for these patients including the frequency and duration of follow-up visits, remain uncertain.^3^ Considering the nature of the disease, it is prudent to incorporate follow-up visits at specific time points for patients with increased natriuretic peptide and normal echocardiogram. These follow-up visits, at a minimum, should adhere to the recommendations for PPCM, including evaluations at discharge from the hospital as well as at 6 weeks and 6 months postpartum. These follow-up assessments provide valuable insights into cardiac function and aid in detecting any changes or potential worsening of the condition. These visits may also be useful in determining the need for long-term diuretic use and use of guidelines-directed medical therapy for HFpEF. Finally, it is also crucial to conduct further studies that focus on identifying specific risk factors or clinical features associated with disease progression and adverse outcomes in PPCM patients. Right heart catheterization data would be crucial in assessing filling pressures and the prevalence of pulmonary hypertension in this disease. Invasive cardiopulmonary exercise testing may also provide important insights into the prevalence of exercise-induced diastolic dysfunction and exercise-induced pulmonary hypertension in this population. Additionally, gaining a better understanding of the long-term outcomes and prognosis for this specific patient population is essential for providing appropriate care and support.

In conclusion, the diagnosis of PPCM poses a challenge, and the ESC study group has offered a valuable diagnostic pathway considering natriuretic peptide levels and LVEF to guide evaluation. However, there are still unanswered questions in the management of patients with elevated natriuretic peptides but preserved ejection fraction. The need for routine follow-up, optimal duration and frequency of follow-up visits, and identification of risk factors for disease progression and adverse outcomes are areas that require further investigation. The lack of specific guidance for follow-up visits, specifically in patients with increased natriuretic peptides and preserved ejection fraction highlights the need for clearer recommendations in this specific patient population. It is important to emphasize the necessity of routine follow-up visits to effectively monitor this condition. Nonetheless, this increased awareness of PPCM and the efforts made by AHA, ACC, HFSA, ESC, and ACOG contribute to improved recognition and understanding of this condition. Further research is crucial to address the existing knowledge gaps and establish evidence-based strategies for the management and follow-up of patients with suspected or diagnosed PPCM, particularly those presenting with an HFpEF phenotype. Moreover, this discussion also underscores the concerning lack of structured cardio-obstetrics training, coupled with the inadequate presence of multidisciplinary cardio-obstetrics teams and substantial knowledge deficiencies among cardiologists. Efforts to enhance cardio-obstetrics education at all career stages are crucial to bridge knowledge gaps and improve patient care in the cardio-obstetric field.^10^

## Funding support and author disclosures

The authors have reported that they have no relationships relevant to the contents of this paper to disclose.

## References

[1] Khan S.S., Brewer L.C., Canobbio M.M. (2023). Optimizing prepregnancy cardiovascular health to improve outcomes in pregnant and postpartum individuals and offspring: a scientific statement from the American Heart Association. Circulation.

[2] American College of Obstetricians and Gynecologists’ Presidential Task Force on Pregnancy and Heart Disease, Committee on Practice Bulletins—Obstetrics (2019). ACOG practice Bulletin No. 212: pregnancy and heart disease. Obstet Gynecol.

[3] Bauersachs J., König T., van der Meer P. (2019). Pathophysiology, diagnosis and management of peripartum cardiomyopathy: a position statement from the Heart Failure Association of the European Society of Cardiology Study Group on Peripartum Cardiomyopathy. Eur J Heart Fail.

[4] Sliwa K., Hilfiker-Kleiner D., Petrie M.C. (2010). Current state of knowledge on aetiology, diagnosis, management, and therapy of peripartum cardiomyopathy: a position statement from the Heart Failure Association of the European Society of Cardiology Working Group on Peripartum Cardiomyopathy. Eur J Heart Fail.

[5] Mehta L.S., Warnes C.A., Bradley E. (2020). Cardiovascular considerations in caring for pregnant patients: a scientific statement from the American Heart Association. Circulation.

[6] Heidenreich P.A., Bozkurt B., Aguilar D. (2022). 2022 AHA/ACC/HFSA guideline for the management of heart failure: executive summary: a report of the American College of Cardiology/American Heart Association Joint Committee on Clinical Practice Guidelines. J Am Coll Cardiol.

[7] Jackson A.M., Macartney M., Brooksbank K. (2023). A 20-year population study of peripartum cardiomyopathy. Eur Heart J.

[8] Tamrat R., Kang Y., Scherrer-Crosbie M., Levine L.D., Arany Z., Lewey J. (2021). Women with peripartum cardiomyopathy have normal ejection fraction, but abnormal systolic strain, during pregnancy. ESC Heart Fail.

[9] Deshmukh A., Kolias T.J., Lindley K.J. (2020). Acute postpartum heart failure with preserved systolic function. JACC Case Rep.

[10] Bello N.A., Agrawal A., Davis M.B. (2022). Need for better and broader training in cardio-obstetrics: a national survey of cardiologists, cardiovascular team members, and cardiology fellows in training. J Am Heart Assoc.

